# Section 377: Why sodomy statutes matter

**DOI:** 10.1002/jia2.25285

**Published:** 2019-05-16

**Authors:** Chris Beyrer

**Affiliations:** ^1^ Desmond M. Tutu Professor in Public Health and Human Rights Department of Epidemiology Johns Hopkins Bloomberg School of Public Health Baltimore MD USA

**Keywords:** sodomy laws, Section 377, decriminalization, human rights, HIV

Institutionalized homophobia, the legal sanction and penalties criminalizing same sex behaviour between consenting adults, remains law in more than 70 countries. While the modern movements for sexual and gender minority rights, the rights of Lesbian, Gay, Bisexual, Transgender, and Queer (LGBTQ) persons, have led to widespread protections in many countries, including marriage equality and parental rights, the gains have been markedly uneven. In Russia, harsh new penalties were imposed in 2013, and have led to brutal attacks against LGBTQ persons, most horrifically in the Russian region of Chechnya 1.

European colonialism left enormously broad and complex legacies across Africa and Asia, North, Central and South America, the Caribbean and the Pacific. Old kingdoms were swept away, peoples were enslaved and transported, new states were created, ancient ones partitioned, and the first true globalized world emerged. For those countries formerly under British colonial rule, Section 377, the colonial statute criminalizing sex between men, and “unnatural” sex between men and women, has remained a challenging social flashpoint, a legal battle ground, and an important domain in the struggle to address HIV and AIDS, particularly among gay, bisexual and other men who have sex with men 2. On the 15th International Day Against Homophobia, Transphobia and Biphobia it is worth unpacking the legacy of Section 377, its uses by homophobic regimes and their supporters, and its lasting importance for the HIV response.

The language of these statutes is archaic and vague, the disdain palpable. Women were largely excluded – except as the sex partners of men in “unnatural” acts – and the widespread misogyny of the time denied women autonomous sexuality. The punishments were severe, from the time of Henry VIII, when “buggery” was first made a civil crime in England (subject to the death penalty) to today.

Here is the language from India's harsh 1861 law that came to be known as Section 377, finally repealed by the Indian High Court in September 2018.

… Unnatural offences: Whoever voluntarily has carnal intercourse against the order of nature with any man, woman or animal shall be punished with imprisonment for life, or with imprisonment of either description for a term which may extend to 10 years, and shall also be liable to fine. 3

In the English‐speaking Caribbean, the British era laws commonly retain the Elizabethan language of buggery, a term then, and sometimes now, used to refer to penetrative anal intercourse, and for hundreds of years a capital offence. Great Britain herself, of course, has moved on: same sex relations were decriminalized decades ago, and Queen Elizabeth II signed the same sex marriage act into law in 2014 4. The UK has pressed for repeal of 377 across the British Commonwealth but the laws persist in more than 40 former colonies and same sex behaviour remains criminalized in more than 70 countries worldwide. Why should these archaic laws matter?

The case of the U.S. may be instructive here. Sodomy statutes were common across the country, particularly in the conservative south, until the landmark 2003 Supreme Court ruling in Lawrence versus Texas, which declared them unconstitutional 5. The decision was based on a 1965 contraceptive rights case which established the right to privacy. In 2003, the Lawrence decision seemed both late in coming and somewhat moot; few if any people had been charged with sodomy for decades in the US. But one of the dissenting justices in the case, the conservative and deeply homophobic Antonin Scalia, saw it differently. This is from his dissent:

Today's opinion dismantles the structure of constitutional law that has permitted a distinction to be made between heterosexual and homosexual unions, insofar as formal recognition in marriage is concerned. 6

Scalia predicted that overturning the sodomy statutes would leave only the social stigma against homosexuality to stand between full rights for LGBTQ persons, including the right to marry. He saw, quite rightly as it turned out, a direct line from decriminalization to marriage equality. The converse is also the case: as long as sex between same sex partners is a crime, the law remains a direct hindrance to any other aspect of social equality and human rights for LGBTQ persons, couples, families and communities. This includes limits on the right to health care.

## 377 AND HIV AND AIDS

The struggles to repeal 377 in the Caribbean, Africa and Asia, have consistently included battles over the impact of these laws on access to HIV care. This became clear in the first successful repeal of 377 in the Caribbean region, the landmark victory of Caleb Orozco versus Attorney General of Belize, in 2016 7. Our Center for Public Health and Human Rights at Johns Hopkins Bloomberg School of Public Health, among other groups, was asked to file a brief to support Orozco's challenge, based on the scientific evidence that the law was a barrier to HIV services. The evidence is clear and consistent that where sexual and gender minority people are subject to criminalization, discrimination and the police harassment that comes from engaging in illegal acts, they are less likely to seek and receive HIV testing, less likely to access pre‐exposure prophylaxis, less likely to be treated for any other sexually transmitted infections, less likely to be able to access treatment if they are living with HIV and to be virally suppressed, and less likely to remain in HIV care 8, 9, 10, 11, 12. The sodomy laws aid and abet the virus, not people and communities at risk.

This seems scientifically straightforward. Yet, it was a contentious issue in the Orozco case, and in other similar cases which challenged 377 statutes in Jamaica, Kenya, and, most recently, Singapore. In each of these court challenges opponents of decriminalization invoked HIV to make a very different argument. One argument (for which there is little or no scientific evidence) goes roughly as follows: “HIV is spread through sex between men, leading to the high HIV rates seen in this population. To control HIV we should use the law to restrict same sex behaviour between men.” This argument was made by a Jamaican physician in support of 377 in the Orozco case 13. Implied in this argument is an embedded theory–also with no evidence behind it – that same sex attraction and activity can be controlled by the state 14. In Kenya, this argument was expanded to suggest that sexual orientation is subject to change.

To be clear, attempts to change sexual orientation, so called “conversion therapy,” have been consistently shown to be both ineffective and harmful, and are associated with higher rates of depression, low self‐esteem, substance use and suicidal ideation among people who have been subjected to these abuses 14. Yet the argument that sodomy laws should remain in place to reduce HIV infection risks persists. In a Kenyan decriminalization case, where a decision was recently delayed and is still pending at this writing, a Kenyan psychiatrist filed a brief opposing repeal on 377 on precisely these grounds.

The repeal of archaic and discriminatory laws from the colonial era is a human rights and social justice struggle. But the strategic litigation against these laws is also an essential part of the effort to expand HIV services and to reach LGBTQ persons and communities who continue to be left behind. If we are serious about achieving the lofty goals of ending the AIDS epidemic by 2030, 377 and all the related laws that continue to criminalize us must be repealed.

## Competing interests

The author has no competing interests to declare.
